# An Evaluation of the Distance at Which Direct Ecological Effects of Released Pheasants Extend Beyond Their Release Sites

**DOI:** 10.1002/ece3.73170

**Published:** 2026-03-04

**Authors:** Joah R. Madden, Maureen I. A. Woodburn, Clive E. Bealey, Joseph L. Werling, Alex N. Banks, Dan Abrahams, Rufus B. Sage

**Affiliations:** ^1^ Center for Research in Animal Behaviour, Psychology University of Exeter Exeter UK; ^2^ Game and Wildlife Conservation Trust Hampshire UK; ^3^ Consulting at Damerham Ltd. Hampshire UK; ^4^ Science Team, Natural England Exeter UK

**Keywords:** ancient semi‐natural woodland, biodiversity, hunting, pheasants, soil nutrients

## Abstract

In the UK, large numbers of pheasants ( *Phasianus colchicus* ) are released into woodlands annually for recreational hunting. Post‐release, they are managed to keep them in areas where they can be shot from October to January. At sites with high pheasant densities, they can negatively impact local flora and fauna through predation, trampling, and altering soil nutrients via defecation. The extent of these effects beyond release pen sites is unclear. This study investigated at what distance from release sites a suite of ecological effects of released gamebirds might be detected. We surveyed along 1 km transects from release pens at 20 shoots in Ancient Semi‐Natural Woodlands (ASNW) in England and Wales. We expected higher soil nutrients, fewer characteristic woodland plants, more nitrogen‐tolerant species, more damage to dead wood, and fewer woody seedlings near pens, with effects decreasing with distance. We found that pheasant numbers encountered declined with distance and sites closer to pens had fewer seedlings/saplings, lower vascular plant richness and less decayed wood (although this last result may be an artefact of the study design). These effects did not extend beyond 500 m. Contrary to expectations, soil nutrients, bare ground, and ancient woodland indicator species showed no consistent change with distance, and nitrogen‐loving species and weeds were more common further from pens. We conclude that, in areas beyond release pens in ASNW in lowland England and Wales, where pheasants are not deliberately enticed, any direct negative effects on plant communities, soil nutrients and ground cover do not extend further than 500 m from the point of release. The current licensing of gamebird releases in England aims to protect designated woodlands by controlling releases occurring within a 500 m buffer around protected areas. Our results suggest that this buffer size appears to be appropriate to contain these effects of released gamebirds.

## Graphical abstract

In the UK, large numbers of pheasants are released into woodlands for recreational hunting, potentially impacting local ecosystems. A survey across 20 sites in Ancient Semi‐Natural Woodlands found that negative effects—such as fewer seedlings, lower plant richness and reduced decayed wood—were mostly confined within 500 m of release pens. Soil nutrient levels, vegetation composition and ground cover showed no consistent patterns with distance from pheasant release pens or differences from control sites.

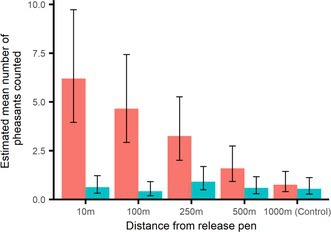

## 1 Introduction

Pheasants *Phasianus colchicus* are released in a number of countries (including USA, New Zealand, Denmark, France, Italy and Czechia) to support recreational shooting (Powolny and Czajkowski 2022; Madden and Sage 2020). In the UK several tens of millions are released annually (Aebischer 2019; Madden 2021). Typically, the birds are artificially reared from day‐old chicks and placed into open‐topped pens in woodland when they are 6–8 weeks old to protect them from ground predators (e.g., red foxes *Vulpes vulpes* ) as they acclimate to independent living (GCT 1996). It is estimated that, in the UK, one in 12 woodlands contain a release pen (Sage et al. 2005), with at least 14% of woodland in the UK managed to some extent for gamebirds, primarily pheasants (Gilbert 2007). In England, this rises to 28% (Gilbert 2007). Therefore, any ecological effects that such releases might have, are likely to be of widespread and general interest. There is particular interest, in the UK, about the potential that these released birds might affect ecologically sensitive areas, particularly those with a conservation designation (Minter et al. 2024).

The direct ecological effects of releasing pheasants can manifest in woodland habitats in two ways. First, the birds themselves exert a series of direct effects on local fauna and flora (reviewed in Madden and Sage 2020, Mason et al. 2020, Sage et al. 2020, 2021, Madden 2023). These can include physical damage through trampling, leading to crushed vegetation and bare ground (Low et al. 2003, Sage et al. 2005, Sage 2018, but see Draycott et al. 2008 and Capstick, Draycott, et al. 2019 who found less bare ground coverage in game managed woods compared to controls). Such damage may also affect resources used by overwintering invertebrates, such as dead wood which can be an important determinant of local biodiversity (Humphrey et al. 2005; Kirby et al. 2005). However, Draycott et al. (2008) found no differences in amounts of deadwood in woodlands managed for pheasants compared to controls. This trampling may also disturb the soil, favouring annual vascular plant species (Sage et al. 2005; Draycott et al. 2008) or reducing coverage by perennials such as ferns (Sage 2018) or the presence of bluebells (Low et al. 2003). This disturbance may lead to a lower establishment of tree seedlings in some regions (Draycott et al. 2008).

A second form of direct effects is soil enrichment through the deposition of nutrients in faeces. The nitrogen (N), phosphorous (P) and potassium (K) remaining from the grains or high protein pellets that the birds have been fed may become concentrated in areas of high gamebird occurrence. Anecdotal reports describe local soil enrichments accompanying large gamebird releases (Alsop and Goldberg 2018, Smith 2014, Rothero 2006—partridges). Controlled comparisons have supported the idea that large numbers of released birds can lead to eutrophication. Sage et al. (2005) found that soil K and P levels were 3 times and 1.5 times respectively higher inside release pens than nearby control areas. Capstick, Sage, and Hoodless (2019) found that these differences might persist for over a decade. However, N levels did not differ between inside and outside pens in these two studies. Likewise, in a study of areas of high‐ and low‐density gamebird releasing (pheasants and red‐legged partridges *Alectoris rufa* ), there were no differences in soil K or N detected between sites (Callegari 2006). These two effects can be measured directly through visual inspection of vegetation coverage, wood availability or through soil chemistry. They can also be detected through the consequent changes in woodland flora.

Soil enrichment, disturbance and disruption of seedling establishment are expected to have a negative effect on ancient woodland indicator (AWI) species (Hermy et al. 1999; Hill et al. 1999). Although AWI species might benefit from increased nutrient levels, more generalist competitor species may benefit even more and so outcompete the AWI species (Hipps et al. 2005). Therefore, we might expect to encounter fewer AWI species or individuals and more annual, ruderal or N‐tolerant species in areas where gamebirds are released. Woodland perennial plants were typically 10%–20% less abundant and weed species 10%–20% more abundant in pens that released more than 2000 or so birds per hectare compared to controls (Sage et al. 2005). When the flora of disused release pens was compared with control sites in the same wood, there was a higher abundance and coverage of species characteristic of high fertility soils, but lower AWI species and overall species richness (Capstick, Sage, and Hoodless 2019). Capstick, Draycott, et al. (2019) reported that, in a different set of woods, on rides (open areas within woodlands, outside release pens) in woods managed for released gamebirds, there was greater quadrat coverage by grass and herbs, and more ruderal species, and species of high soil fertility and more overall species, but not higher numbers of AWI species, compared to rides in woodland not managed for game.

Negative ecological effects are expected to be greatest in areas of high densities of released pheasants (reviewed in Madden and Sage 2020, Mason et al. 2020, Sage et al. 2020). Consequently, previous work has predominantly focused on measuring such effects within the release pens themselves. However, the birds can disperse from these pens and therefore there is a risk that negative ecological effects might extend beyond these immediate areas. Dispersal of released gamebirds is unlikely to be random. Game managers deploy habitat management and feeding strategies that largely restrict movements and retain the released birds on the surrounding land over which they have permission to shoot, and to direct them to particular areas of cover from where they can be flushed (driven into flight) during a shooting day (GCT 1996). Therefore, it is likely that most released pheasants remain close to the pens. The largest study of movement of released pheasants on managed shoots (Turner 2007) revealed that the overall average maximum distance that a pheasant moved from the pen was 913 m and just under 90% of the tracked pheasants had a home range of ~280 ha or less, which would equal a circle with a radius of 940 m (Sage et al. 2021). A number of smaller studies, including some conducted in the breeding season after release and outside the UK, reported dispersal distances using a range of different metrics as being a few hundred metres (reviewed in Sage et al. 2021). Therefore, the numbers of pheasants being found far from their release pen may be relatively small. However, given the large numbers of birds being released, the absolute numbers may still be noticeable. In early spring after the shooting season has ended, densities of pheasants range from 100 to 450/km^2^ at release sites, and 20–60/km^2^ at non‐release sites situated 0.5–2.5 km from managed areas (Sage et al. 2025). Densities, of course, decrease spatially with distance as the area available increases following a power function (Sage et al. 2021). Densities also decrease temporally as mortality rates, both natural and due to hunting, are high, with perhaps just 9%–15% of the released birds still alive at the end of the shooting season (Madden et al. 2018).

Given the previous focus of research in this area on the release pens themselves, coupled with the uncertainty about where dispersed pheasants might move to and survive, it is unknown to what distance these effects might extend and so what area of woodland might be affected by the direct effects of released pheasants. Previous studies have compared measures made within the release pen to sample sites at a variety of distances away (Pressland 2009—adjacent to, but outside, the pen; Hall et al. 2021—25 m away; Sage et al. 2005—50 m away; Capstick, Sage, and Hoodless 2019—100–150 m away; Sage 2018 > 100 m away; Neumann et al. 2015 250–300 m away). This variation in sampling distance makes direct comparisons difficult. In this study, we deliberately investigated the distance at which any effects might extend outside the pen by conducting transects leading out from the release pen for 1 km, along which we expected a decay in any effects might be detected. At five survey points along the transects at 10 m, 100, 250, 500 and in a control plot at least 1000 m away, we measured a set of indicators of direct physical damage (decayed wood availability, bare ground coverage and seedling establishment); a set of soil nutrient indicators (bio‐available soil nitrates, phosphates and potassium levels); and a set of floral measures (overall flowering plant species richness, AWI species abundance, weed abundance, and Ellenberg's N community composition). We tested at what distance these indicators differed from measures just outside the release pen (when we could conclude that the effects of the released birds had declined) and at what distance these indicators differed from the furthest point, at least 1000 m from the release pen and usually in a separate block of woodland (which we took as being a control site where the released pheasants had no or very limited effects). We confirmed the densities of pheasants at different distances by counting them at the survey sites. We conducted pheasant surveys in both the winter, when pheasant numbers were expected to be high and concentrated around the release pen, and in early summer when most released pheasants were either dead or no longer under the direct management of gamekeepers and so expected to move freely.

We predicted that we would encounter relatively fewer pheasants at sites further from the release pen. Consequently, we predicted that higher densities of pheasants close to the pen would mean that soil nutrient levels, bare ground coverage, plant communities with higher Ellenberg N scores and abundance of weed species would all be higher close to the pen. Conversely, the availability of decayed wood, the presence of germinating seedlings, and the abundance of Ancient Woodland Indicator plant species (and perhaps overall vascular plant species abundance) would all be higher further from the pen.

## 2 Methods

### 2.1 Study Sites

We worked at 20 sites, 18 in England and two in Wales (Table 1). Sites were selected because they: were blocks of broadleaf ancient semi‐natural woodland (ASNW), some of which were also designated Special Areas of Conservation (SAC) or Sites of Special Scientific Interest (SSSI); contained a large pheasant release pen from which 600–8000 pheasants were released annually and managed by a professional keeper (these sizes of releases represent 52% of shoots recorded in the APHA Poultry Register Madden and Sage 2020 and align with the shoots studied in Sage et al. 2025 which deliberately sort out representative shoots); were large enough to accommodate a 500 m transect (we attempted to find sites that could host a continuous 1 km transect, but these were very scarce); if we couldn't find a wood large enough to accommodate the full transect, we selected a control wood of the same composition as the pen wood 1–3 km away, deliberately avoiding differences in species, age, and structure, to associate with the pen wood; permitted access to researchers and provided (some) information about release numbers. Site owners were assured of anonymity, hence the reporting of site positions at the resolution of county. We note that our study may not represent patterns at small/amateur shoots where there is no professional game management and where, due to the small release sizes, densities are low.

**TABLE 1:** The distribution of 20 study sites by county across England and Wales.

County	Number of sites
Cambridgeshire	1
Cornwall	2
Cumbria	1
Denbighshire	1
Devon	2
Hampshire	3
Herefordshire	1
Kent	2
Leicestershire	1
Northamptonshire	1
Powys	1
Somerset	1
Staffordshire	1
Wiltshire	2

### 2.2 Survey Design

At each site, we started our transect at the release pen. Previous studies have measured a suite of indicators of ecological effects within the pen (Hall et al. 2021, Capstick, Sage, and Hoodless 2019, Sage et al. 2005, Pressland 2009, Neumann et al. 2015). Therefore, our first sample plot was 10 m outside the pen, and then samples were made at 100, 250 and 500 m, and at a control plot 1000–3000 m away. The first four plots were all within the wood containing the releasing pen and passed through areas where no focussed game management occurred (feeding sites, game cover planting). The control plot was sited in the same wood where possible, but when the release pen woodland block was too small, we selected a neighbouring wood that was not game‐managed but was similar to the release pen wood in terms of tree species and age as far as possible. The transect was usually in a straight line which varied in orientation between sites according to the shape of woodland and location of the releasing pen within it. The position of each plot was determined using a handheld GPS device with accuracy of 5–10 m depending on canopy cover. In some cases, plots were not situated at exactly these distances due to local conditions for example, tracks, felling patches, etc. In such cases, we surveyed at the nearest area where woodland was complete and representative. The main surveys of ecological variables were conducted between April and June 2024. At each plot, we concentrated on five points. These comprised a central point and four satellite points positioned at 90° intervals around the central point, 15 m away. We placed a 1 × 1 m quadrat in the centre of each point, and recorded ground flora and bare ground coverage within it using the DAFOR scale (Dahl and Hadac 1941; Hill 2005) and the presence or absence or seedlings/saplings. The simple five‐point DAFOR system was used to facilitate a rapid relative assessment and the basis for a mean percentage coverage variable (rare = 3; occasional = 10; frequent = 23; abundant = 40; dominant = 75) that was suitable for comparing plots within and between sites. We surveyed an area of 6 m radius around each point to calculate the amount of decayed wood and abundance of flowering plants in flower.

We collected five sets of data at each plot.

- Gamebird counts: We used a basic non‐vehicle protocol (Sage et al. 2025). This involved recording all pheasants seen or heard during the rest of the habitat assessment and while moving to the next plot. This produced a relative index of encounters per hour of survey at each plot which could be compared between plots and sites. Gamebird counts were made in both the main survey visit and a one‐day survey at each site between Nov 2023 and Feb 2024 to reveal winter gamebird numbers.
- Soil samples: At each plot, we collected five soil samples, one from each quadrat after ground cover had been assessed. Samples were collected using a metal Grass Plot Sampler (NHBS Ecology supplies), providing a volume of 35 mL of soil at a depth of 7.5 cm. The samples from each plot were combined and mixed. The samples were stored in the dark and cold before processing. Processing entailed mixing the samples from a single plot by hand in a single plastic bag to ensure uniformity, removing ~2.5 g of fresh soil for nitrate analysis (kept at 5°C), and then drying the remainder of the sample at 50°C for 60 h until constant mass was attained. Samples were then sieved using a 2 mm mesh. Soil nutrient analysis was conducted under contract in the Department of Geography, University of Exeter. Details of their protocols are provided in ESM1. They supplied us with measures of nitrates, phosphorus, and potassium for each plot. The detected levels spanned: Nitrates, 0.0018–0.076 mg/g soil; Phosphates, 0.00063–0.63 mg/g soil; Potassium, 0.73–8.01 mg/g soil.
- Decayed wood availability. We estimated the volume of decayed wood within the 6 m radius circle around each point using a DAFOR score. All cut/felled wood was excluded from this, so it included only naturally fallen or otherwise dead/decaying wood. Where available, three pieces of dead wood were randomly selected using pacing and turning and assigned a decay class (1–5) based on forestresearch.gov.uk guidelines, where 1 = little decay, bark hard and intact to 5 = extensive decay, bark absent and wood texture very soft and spongy. For each plot, we multiplied the DAFOR score by the mean decay class for the point to obtain a single measure combining quantity and quality per point. Together, these metrics provided a crude metric but one that summarised the availability of decayed wood that might be suitable for shelter/larval deposition sites for invertebrates. For example, a high score would indicate that there was lots of well‐rotted wood, making it highly suitable for invertebrates.
- Seedling count. We recorded simply whether there were any germinating tree or other woody plant seedings present in the 1 m^2^ quadrat at each point, scoring 1 for presence and 0 for absence. There were very seldom fewer than one germinating tree/woody plant seedling in any quadrat, so this presence absence metric provided a realistic representation of occurrence.
- Flowering plant diversity and abundance. All vascular plants within the quadrat at each point were identified and their coverage scored using a DAFOR scale. We also scored the coverage of bare ground using the same scale. For each species, we classed it as an Ancient Woodland Indicator (AWI) species, based on Peterken (2000), or a weed species based on SSSI Condition Monitoring Guidance JNCC (2004), or neither (for full lists of species included in each group, see ESM2). From these classifications, we could calculate for each point the coverage by weed species (summing the DAFOR scores for all weed species in the quadrat) and the coverage by AWI species present (summing the DAFOR scores for all AWI species in the quadrat). For each species, we also obtained its Ellenberg N score (Hill et al. 1999), ranging from 1 to 9, with high scores indicating extremely nitrogen‐rich soils such as cattle resting places or polluted rivers. We derived the mean Ellenberg N score for all the plant species in each quadrat to provide one measure per point. We calculated species richness for each quadrat by counting the number of unique species recorded in it. Finally, we obtained a DAFOR score for bare ground cover within each quadrat.

Therefore, for each plot, we had two measures of gamebird abundance, one soil sample (for nitrate, phosphate and potassium levels), and five measures each of decayed wood availability, sapling/seedling presence, AWI species coverage, weed species coverage, bare ground coverage, average community N tolerance and vascular plant richness.

### 2.3 Analyses

Analyses were conducted using mixed models to account for the use of repeated transect measures across multiple study sites. The general structure was to include the five distance‐from‐the‐pen categories as the main independent variable of interest with additional variables included as necessary with interactions where appropriate (Table 2). This allowed us to ask two questions in each test. First, did the measure collected just outside the pen differ from that collected at the control site, > 1 km away from the pen in a woodland where no gamebird management was conducted? Second, at what distance (100, 250, 500, 1000 m) could we detect any differences from the measure collected just outside the release pen? We addressed these questions by conducting post hoc contrast tests using the emmeans package (Lenth 2025) with Tukey correction for multiple comparisons. A generalised model structure was used because all dependent variables were counts or proportions with error structures and link functions selected as appropriate to the data (Table 2). Some variables were highly skewed. We checked measures of dispersion and zero inflation using the R package *DHARMa* (Hartig 2022). Where necessary, we adjusted error structures and/or used zero inflated model approaches to ensure model assumptions were met (Table 2). We inspected model fit visually. *p* values of main effects or interactions were derived using the ‘drop1’ function. Although we tested the effects of 11 dependent variables and thus made multiple comparisons of the same question (e.g., at what distance category does variable X show differences from measures just outside the pen), this was an exploratory study, and we did not adjust the alpha value for initial analyses. Therefore, conclusions about individual variables should be made with care for *p* < 0.0045. Full model outputs are given in ESM3. All analyses were conducted using the R package *glmmTMB* (Brooks et al. 2017) in R (R Core Team 2024).

**TABLE 2:** Summary of generalised mixed model structures used.

	Main variable of interest	Any co‐variates?	Random terms included	glmmTMB family	Link function used	Zero‐inflated model used
Gamebirds	Count of gamebirds	Season (summer/winter) Count × Season	Site	nbinom2	Log	No
Nutrient levels	Soil nitrates	None	Site	Gaussian	Identity	No
	Soil potassium	None	Site	Gaussian	Identity	No
	Soil phosphates	None	Site	Gaussian	Identity	No
Physical disturbance	Bare ground coverage	None	Site	nbinom2	Log	Yes
	Presence of tree seedlings	None	Site	binomial	Logit	No
	Availability of decayed wood	None	Site	gamma	Log	Yes
Ecological indicators	Ellenburg's N community composition	None	Site	Gaussian	Identity	No
	Ancient Woodland Indicator species abundance	None	Site	nbinom2	Log	Yes
	Weed abundance	None	Site	nbinom2	Log	No
	Species Richness	None	Site	Poisson	Log	No

## 3 Results

### 3.1 Do the Numbers of Gamebirds Detected Differ Depending on Distance From Release Sites?

**FIGURE 1:**
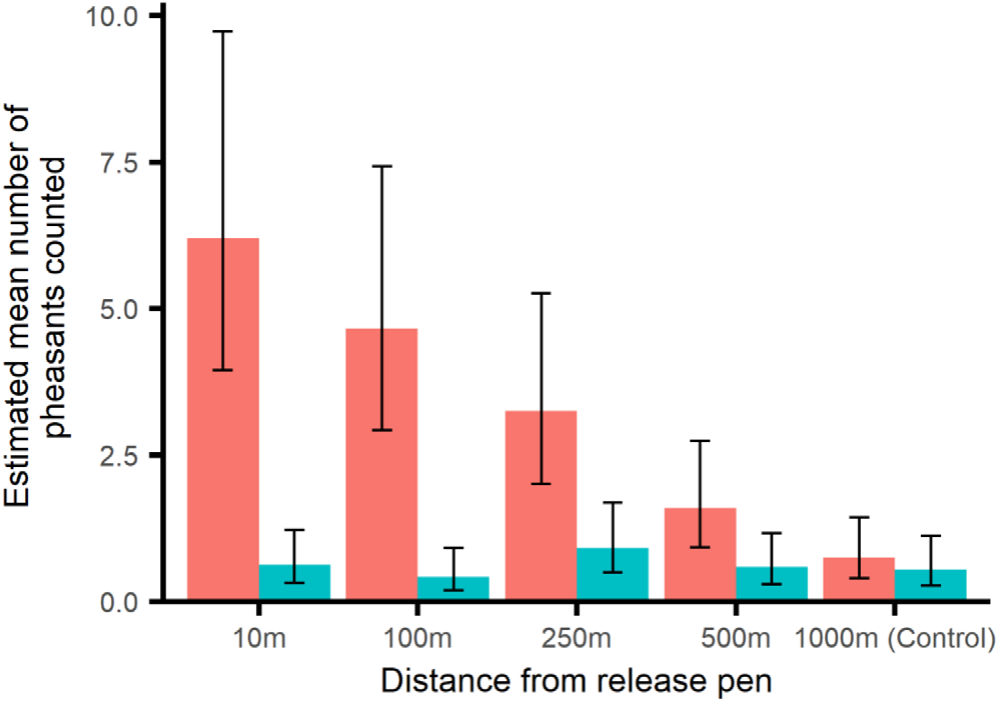
Estimated mean number of pheasant encounters at five distances from woodland release pens during winter (red) and summer (blue) surveys. Error bars indicate 95% CI.

The effect of distance from release pen on the number of pheasants encountered during surveys differed between the winter and summer (Distance × Season: *χ* ^2^_4_ = 18.5, *p* = 0.001, ESM3–1, Figure 1). During the winter, the number of pheasants declined with distance from the pen, but during the summer there was no such relationship, and the absolute numbers detected were lower than during the winter. There were 8.2× fewer pheasants detected at the control site compared to just outside the pen (post hoc contrast, *p* = 0.0011). Numbers of pheasants detected at the control site did not differ from winter to summer.

### 3.2 Do Soil Nutrient Levels Differ Depending on Distance From Release Sites?

#### 3.2.1 Nitrates

Levels of bio‐available nitrates did not differ depending on survey distance from the release pen (*χ* ^2^_4_ = 4.13, *p* = 0.41, ESM3–2a). The levels just outside the pen did not differ from the control sites (post hoc contrast, *p* = 0.98). There were no differences in levels just outside the pen and any of the other four distance measures (post hoc contrast, all *p* > 0.72).

#### 3.2.2 Phosphates

Levels of bio‐available phosphates did not differ depending on survey distance from the release pen (*χ* ^2^_4_ = 2.38, ESM3–2b, *p* = 0.67). The levels just outside the pen did not differ from the control sites (post hoc contrast, *p* = 0.99). There were no differences in levels just outside the pen and any of the other four distance measures (post hoc contrast, all *p* > 0.85).

#### 3.2.3 Potassium

Levels of bio‐available potassium did not differ depending on survey distance from the release pen (*χ* ^2^_4_ = 7.44, ESM3–2c, *p* = 0.13). The levels just outside the pen did not differ from the control sites (post hoc contrast, *p* = 0.26). There were no differences in levels just outside the pen and any of the other four distance measures (post hoc contrast, all *p* > 0.26).

### 3.3 Does Bare Ground Cover Differ Depending on Distance From Release Sites?

The area of bare ground in a quadrat (numerised DAFOR score) did not differ depending on survey distance from the release pen (*χ* ^2^_4_ = 7.44, ESM3–3, *p* = 0.80). The levels just outside the pen did not differ from the control sites (post hoc contrast, *p* = 1.0). There were no differences in levels just outside the pen and any of the other four distance measures (post hoc contrast, all *p* > 0.87).

### 3.4 Does the Presence of Germinating Seedlings and Saplings Differ Depending on Distance From Release Sites?

**FIGURE 2:**
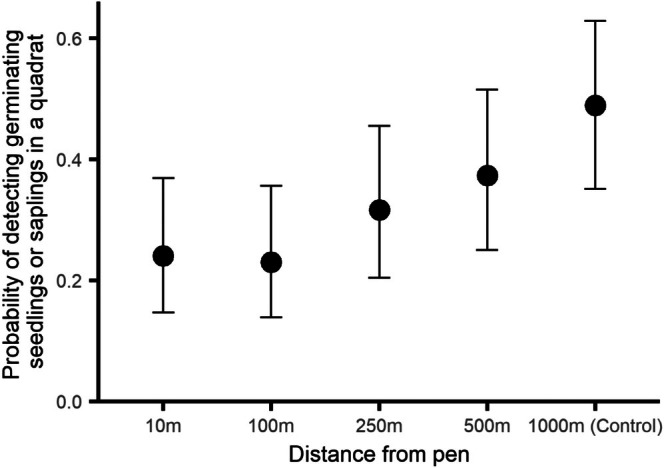
The probability of detecting woody tree or shrub seedlings or saplings in a quadrat at five distances from woodland pheasant release pens during summer surveys. Error bars indicate 95% CI.

The probability of detecting germinating tree and shrub seedlings and saplings in a quadrat differed depending on the distance of the quadrat from the release pen (*χ* ^2^_4_ = 17.45, ESM3–4, *p* = 0.0013, Figure 2). There was a greater probability of detecting them in the control site compared to just outside the release pen (post hoc contrast, *p* = 0.006), but probabilities at other distances did not differ from those just outside the release pen (post hoc contrasts, all *p* > 0.30).

### 3.5 Does the Availability of Decayed Wood Differ Depending on Distance From Release Sites?

**FIGURE 3:**
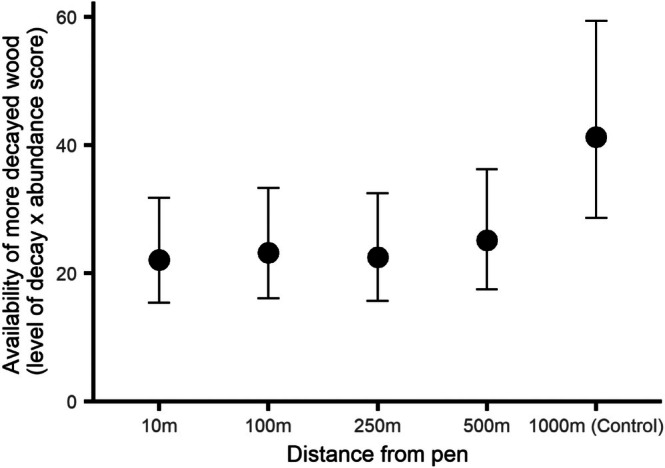
The availability of decayed wood (calculated by multiplying the mean level of decay of the wood recorded by the DAFOR abundance score) at five distances from woodland pheasant release pens during summer surveys. Error bars indicate 95% CI.

The availability of more decayed wood differed depending on the distance of the survey point from the release pen (*χ* ^2^_4_ = 21.5, ESM3–5, *p* = 0.0001, Figure 3). There was more available in the control site compared to just outside the release pen (post hoc contrast, *p* = 0.0008), but quadrats at other distances up to this point did not differ from those just outside the release pen (post hoc contrasts, all *p* > 0.92).

### 3.6 Does the Average Plant Community N Sensitivity in a Quadrat Differ Depending on Distance From Release Sites?

**FIGURE 4:**
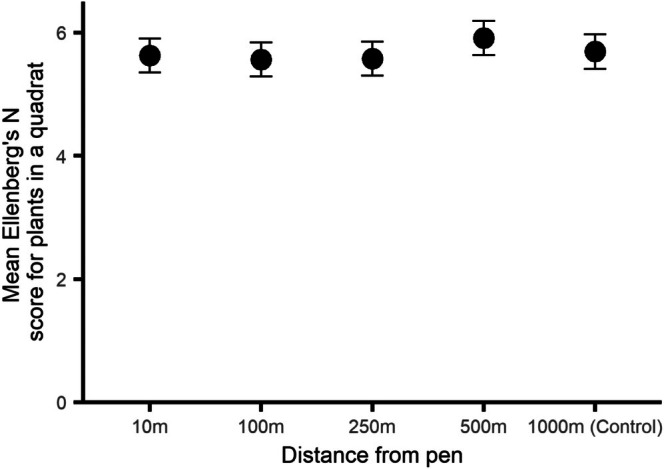
The mean Ellenberg's N score for plant species recorded in quadrats at five distances from woodland pheasant release pens during summer surveys. Higher values indicate that the species prefer more nutrient‐rich soils. Error bars indicate 95% CI.

The mean Ellenberg N score for plant species recorded in a quadrat differed depending on the distance of the survey point from the release pen (*χ* ^2^_4_ = 22.2, ESM3–6, *p* = 0.0002, Figure 4). The mean score at the control site did not differ from that just outside the release pen (post hoc contrast, *p* = 0.95), but quadrats at 500 m had a higher mean score (species favouring more nutrient rich soil) than those just outside the release pen (post hoc contrast, *p* = 0.0083).

### 3.7 Does the Abundance of Ancient Woodland Indicator Species Plants Differ With Distance From Pen?

There was no difference in the abundance (sum of numerised DAFOR scores for all AWI plants in a quadrat) of Ancient Woodland Indicator plant species recorded in quadrats with distance from the release pen (*χ* ^2^_4_ = 3.41, ESM3–7, *p* = 0.51). The abundance scores from quadrats just outside the pen did not differ from the control sites (post hoc contrast, *p* = 0.58). There were no differences in abundance scores from quadrats just outside the pen and any of the other four distance measures (post hoc contrast, all *p* > 0.58).

### 3.8 Does the Abundance of Weed Species Plants Differ With Distance From Pen?

**FIGURE 5:**
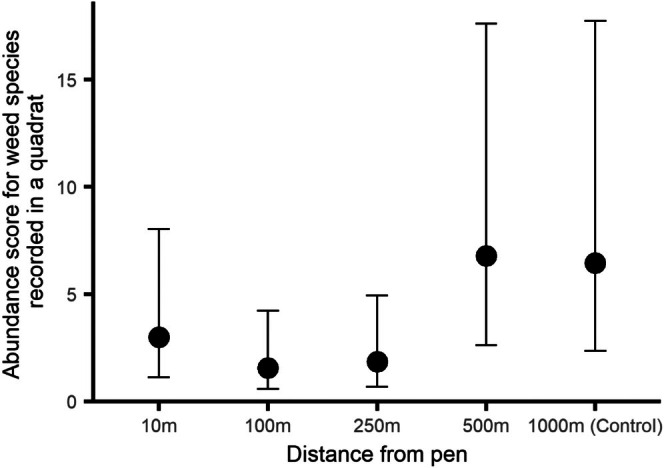
The abundance score (sum of numerised DAFOR scores for each weed species) for weed species recorded in quadrats at five distances from woodland pheasant release pens during summer surveys. Error bars indicate 95% CI.

The abundance (sum of numerised DAFOR scores for all weed species in a quadrat) of weed species recorded in quadrats differed with distance from the release pen (*χ* ^2^_4_ = 14.6, ESM3–8, *p* = 0.0060, Figure 5). However, there was no difference in the abundance scores from quadrats just outside the pen and the control sites (post hoc contrast, *p* = 0.58), nor were there any differences in abundance scores from quadrats just outside the pen and any of the other four distance measures (post hoc contrast, all *p* > 0.39). There were generally more weeds in quadrats at 500 and 1000 m compared to quadrats closer to the release pen.

### 3.9 Does the Total Number of Species of Vascular Plants Differ With Distance From Pen?

**FIGURE 6:**
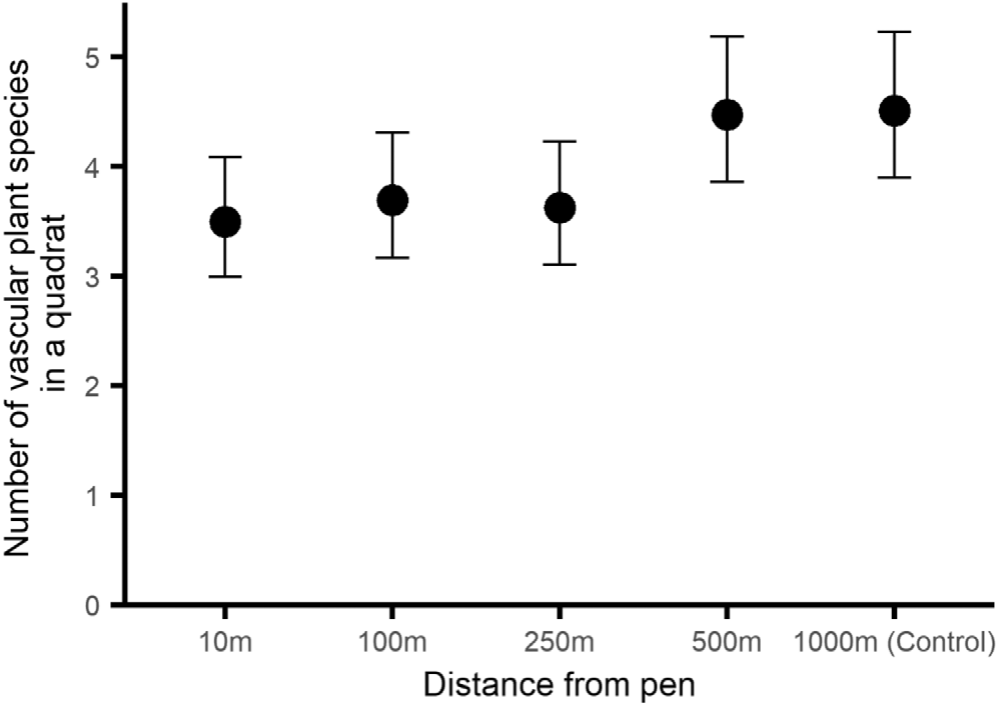
The mean number of vascular plant species recorded in quadrats at five distances from woodland pheasant release pens during summer surveys. Error bars indicate 95% CI.

The number of species of vascular plants recorded in a quadrat differed with distance from the release pen (*χ* ^2^_4_ = 25.2, ESM3–9, *p* < 0.0001, Figure 6). There was approximately one more species recorded in quadrats at 500 and 1000 m (mean = ~4.5 species) compared to quadrats closer to the release pen (mean = ~3.5 species) (all post hoc contrasts between 500 & 1000 m and 10–250 m, *p* < 0.043). There was no difference in the numbers of species recorded between quadrats at 10–250 m (all post hoc contrasts, *p* > 0.94) or in numbers of species recorded between quadrats at 500–1000 m (post hoc contrast, *p* = 0.99).

## 4 Discussion

Evidence for direct negative ecological effects caused by soil eutrophication and the physical actions of pheasants at distance outside their release pen is mixed (Table 3). Our predictions about increased distance from the release pen corresponding to decreases in pheasant abundance (in winter) and increases in the presence of seedlings and decayed wood were supported, suggesting that released pheasants may cause these sorts of physical disturbance outside release pens. Our weak prediction that overall vascular plant species richness increased with distance from the pen was also supported, but this richness may depend on weed species which we expected to be higher around the pen. However, we could not support our predictions that soil nutrients, bare ground coverage or Ancient Woodland Indicator species differed with distance from the pen, suggesting that pheasants may not be responsible for these anticipated effects. We also found that, contrary to our predictions, more N tolerant plant communities and weed abundance increased further from the pen. The abundance of pheasants in the winter declines as the distance from their release pen increases. In the summer this trend no longer holds, but the overall number of encounters is much less. This makes sense given the usual pattern of release management and shooting (GCT 1996) and justifies our hypothesis that their direct ecological effects affecting the ground flora might also decline with distance from their release pen. We found some evidence that the number of established tree seedlings and saplings, and the availability of decayed wood, both of which may be susceptible to physical damage by foraging gamebirds, were higher at sites > 500 m from the release pen compared to sites closer to the pen. There was also a higher richness of vascular plant species further from the pen with plots > = 500 m having the highest richness. However, the species in these plots had a higher mean Ellenberg's N score, indicative of species favouring more nutrient rich soils, and this was accompanied by generally more coverage by species that respond to this nutrient (often classed as weeds) at these more distant plots. We found no evidence of changes in coverage by AWI species as distance from the pen increased, nor any change in bare ground cover. We found no evidence of declines in soil nutrients (nitrates, phosphates and potassium) that might be attributable to reduced faecal depositions as distance from the release pen increased and gamebird numbers declined.

**TABLE 3:** Summary of results evaluating the hypothesis that the direct ecological effects of released pheasants decline with distance from woodland release pens.

	Main variable of interest	Any overall effect of plot location	Was there a difference in the main variable between the pen and control plots?	At what distance did the main variable first differ from measures at the release pen, and in what direction?
Gamebird abundance	Gamebird abundance	Yes	Yes	250 m in winter, with 54% of pheasants compared to immediately outside the pen
Nutrient levels	Soil nitrates	No	No	N/A
	Soil potassium	No	No	N/A
	Soil phosphates	No	No	N/A
Physical disturbance	Bare ground coverage	No	No	N/A
	Number of tree seedlings	Yes	Yes – more seedlings at control sites	1000 m/Control site = more seedlings at 1000 m/control sites—supports prediction
	Availability of decayed wood	Yes	Yes—more decayed wood at control sites	1000 m/Control site = more decayed wood at 1000 m/control sites—supports prediction
Ecological indicators	Ellenburg's N community composition	Yes	No	500 m = higher mean Ellenberg's N score (species favouring more nutrient rich soil—opposite to prediction) but difference lost at 1000 m/control sites
	Ancient Woodland Indicator species abundance	No	No	N/A
	Weed abundance	Yes	No	No difference from outside release pen, but generally more weeds at 500 m and 1000 m/control—opposite to prediction
	Species richness	Yes	Yes	500 m = higher species richness compared to outside pen—supports (some) predictions

Numbers of gamebirds in woodland surrounding release pens is highest in the winter, during the shooting season, but falls markedly at all distances from the pen in the spring/summer. We note that we did not collect any data in the areas deliberately managed and fed near to pens, which most dispersing released birds would be expected to occupy. At the control plot 1–3 km from the pen, numbers of gamebirds recorded in the winter were similar to those seen spread evenly throughout the woodland in summer, with both these indices being about eight times lower than the number recorded immediately outside the pen during winter. This confirms that the likely direct effects attributable to released pheasants are expected to decline with distance from release sites and also be markedly lower during spring/summer. Our measures of gamebird abundance were indices and difficult to translate into absolute numbers, because exact release numbers were not available, survey dates varied and mortality rates to these survey points are likely to be variable. Estimates by Sage et al. (2025) suggest that at a suite of professionally managed shoots in southern England, there might be 100–450 pheasants/km^2^ at the end of the shooting season in land around the release pens, with 100 pheasants/km^2^ in early summer. Regardless of absolute numbers, our indices confirm that any effects occurring due to the direct actions of pheasants that occur during the winter (e.g., nutrient deposition, damage to decayed wood, and disturbance of the ground) are expected to decline with distance from the pen. Direct effects that occur during the summer are expected to be markedly lower than any effects occurring during the winter, because there are far fewer birds still alive, but are unlikely to differ with distance from the pen because the few remaining birds by this time are spread more evenly through the woodland.

Pheasants are predominantly terrestrial omnivores and commonly scrape at the ground and materials to access plants, seed and invertebrates (Hill and Robertson 1988). Our findings that there was a greater probability of finding seedlings or saplings at the control site compared to immediately outside the release pen, and a general increase at plots further from the pen, support the prediction that the direct actions of released pheasants might reduce sensitive woody seedlings and saplings outside release pens, perhaps to distances of 500 m. This effect was detectable despite the very crude reliance on presence/absence of any germinating seedlings in each quadrat. Draycott et al. (2008) also found tree‐seedling numbers were lower in game‐managed wood compared to controls. Physical disturbance of seedlings or saplings was not matched by evidence of disturbance of decayed wood. We found no effect of distance to pen on decayed wood within and including 500 m of the pen. There was more decayed wood in the control wood, so we cannot rule out an effect of pheasants. However, the pheasant index trend and the decayed wood data up to and including 500 m suggests that pheasants themselves were not having an effect while the comparison between all other plots with the non‐release control wood suggests there may be an effect of management. Good game woods generally involve management to provide open canopy areas and woody cover (e.g., Robertson et al. 1993) and there tends to be an increase in these features in these woods and the management actions that produce them (Rackham 2003; Oldfield et al. 2003). We did not include cut timber when assessing deadwood in our study and more management of trees leads to less naturally decaying wood. Draycott et al. (2008) found no differences in deadwood availability between woods managed for gamebirds compared to controls. We also found no evidence that their scraping resulted in larger areas of bare ground, with no pattern in coverage varying with distance, this suggests that the detectable effects on bare ground reported by Low et al. (2003), Sage et al. (2005) and Sage (2018) are restricted to within the release pen, and it matches findings by Draycott et al. (2008) who reported no overall differences in bare ground cover between game‐managed and control woods. Disturbed bare ground might be rapidly colonised by annual pioneer species meaning that any effects are ephemeral (e.g., Grime 2006).

We found no evidence that soil nutrient levels consistently changed with distance from the release pen. For each of nitrates, phosphates and potassium, levels were higher (but not significantly so) in the control plots than immediately outside the pen. This suggests that the high and persistent in‐pen nutrient levels reported by Sage et al. (2005) and Capstick, Sage, and Hoodless (2019) are the product of the high densities of birds concentrated in the pens early in the season. The absence of an effect over a larger scale matches that reported by Callegari (2006) who found no differences between overall sites where high or low densities of gamebirds had been released. Obvious point sources of nutrient enrichment (e.g., pig or chicken units or manure heaps) were avoided as far as possible during our site selection phase but the furthest points on the transects were sometimes nearer arable or grass fields or roads than the actual release pen points. It is possible that our survey plots further from the release pen were sometimes exposed to greater atmospheric deposition of nutrients onto the soil than plots near to the pen, potentially reducing or blurring the effect of the pen itself.

Our measures of vascular plant species indicators showed mixed support for the prediction that released pheasants exert direct negative ecological effects on these at distance from the pen. Previous work has shown that plant community diversity and composition within pens, including AWI and overall species richness, is lower compared to control areas (Sage et al. 2005; Capstick, Sage, and Hoodless 2019), attributing differences to eutrophication, shade levels and soil disturbance. Comparisons of control sites with game‐managed sites away from the release pens have reported mixed results. Sage (2018) did not find differences between control and game woods in SW England. In a remote sensing study covering Great Britain, no overall differences in plant species density were reported (Long et al. 2025). In a comparison of rides in woods in S England, there were more ruderal species, and species of high soil fertility and more overall species, but not higher numbers of AWI species in game‐managed woods compared to controls (Capstick, Draycott, et al. 2019). Overall, we found there was a higher species richness of vascular plants > 500 m from the release pen and richness at our control plots was higher than immediately outside the pen. This might indicate that pheasants cause direct damage to plants such that only a subset can thrive. However, alternative reasons are also possible. We suspect that factors such as distance to woodland edge or proximity to other sources of disturbance or pollution (that the pen may have been deliberately sited away from) could also explain these patterns. This increase in habitat diversity or proximity to the woodland edge might also explain the increase in vascular plant species diversity at 500 m + from the pen. We found that at plots 500 m from the release pen, plant species recorded there were, on average, those that favoured more nutrient (nitrogen)‐rich soil. This was despite our finding of no effect of distance from the pen on soil nutrient levels, suggesting that factors other than pheasant defecation, or pheasants at all, might be responsible for this pattern. We also found that coverage by weed species increased at plots > 500 m from the release pen. However, this result was not significant when we corrected alpha values to account for multiple testing (*p* = 0.0045), indicating that this unexpected relationship should be treated with caution. We expected annual, ruderal species to thrive in areas disturbed by pheasants, but our findings directly contradict this. Coverage by AWI species showed no change with distance from the release pen, suggesting that at least for these characteristic woodland species, the actions of pheasants did not affect them. Consequently, we found little consistent evidence that physical damage or eutrophication by released pheasants led to detectable changes in vascular plant communities at distances beyond the release pens.

Our study design and interpretation of the results was underpinned by the assumption that any differences between plots in ecological measures according to distance from the pen were dependent on the actions of the dispersing pheasants. However, it was difficult to control for other variables across our sample of 20 sites. Most obviously, while the numbers of birds released at all sites exceeded 600, across the 20 sites the number probably differed by an order of magnitude. However, we did not intend to, and as it turned out could not, include this variable in our analyses because at 20% of our sites the keepers did not want to report release numbers and at others their accuracy was suspect. In addition, previous work has found that ecological effects of game management differ across regions of England (Draycott et al. 2008; Heydon and Reynolds 2000; Woodburn and Sage 2005; Sage et al. 2009) making national conclusions difficult. We tried to make our findings generalisable across lowland England and Wales and so conducted sampling in a wide range of regions, albeit restricted to broadleaf ASNW. Effects that might manifest in one region due to particular soil types or native plant communities might be absent under other conditions, adding noise to our models and obscuring local effects. Our attempts to match control sites to managed woodlands at specific distances were likely imperfect. Despite our attempts to match species composition, stand ages, management actions and block size (qualitatively assessed during an initial visit), it was seldom possible to obtain perfect matches. However, we believe that there was no bias in any particular dimension, so the control woods represent a reasonable comparator group. Finally, pheasant release pens are usually situated non‐randomly within woods, typically being near to a woodland edge, and in areas that offer a natural mix of habitat types to provide shelter, sunning and roosting opportunities for the birds in them (GCT 1996), or in areas away from public access to reduce human disturbance, or in areas that are easy for keepers to access. Therefore, transects leading from them, deliberately selected to remain within the same woodland for at least 500 m, may have crossed into very different habitats, perhaps going deeper into the woods where shading and microclimates alter plant communities, or towards woodland edges, or towards areas where humans and dogs might create disturbance. The patterns that we observed, including the unexpected higher weed coverage and N‐tolerance further from the pen, may be due to factors other than the direct effects of pheasants acting at a decreasing level with increasing distance from the release pen.

Currently, a licencing process exists in England to reduce potential direct negative ecological effects from released gamebirds on some protected areas. Releases within Natura 2000 sites (Special Areas of Conservation‐SACs; Special Protection Areas—SPAs) and within a 500 m buffer zone around those sites require a licence, the exact type depending on designation and release densities (https://www.gov.uk/guidance/gamebirds‐licences‐to‐release‐them#:~:text=If%20your%20planned%20release%20is,release%20gamebirds%20in%20other%20areas). The basis for the current buffer zone is a review of the literature conducted by Madden and Sage (2020) which crudely evaluated distances that released gamebirds might disperse. However, they did not explicitly consider the ecological effects that may arise from the birds at this distance, given their diffusion into larger areas and their mortality as the season progressed. Our work provides evidence to inform decisions about future licencing. Our surveys of gamebird abundance indicate at the middle to end of the shooting season, by which time at least half the released birds are already dead (Madden et al. 2018), around one fifth of the surviving birds are found more than 500 m from the release pen (but away from the core game managed and fed areas). Numbers of seedlings/saplings, amount of decayed wood and vascular plant richness may be lower up to 500 m from the release pen. If these effects are attributable to the direct actions of released pheasants, then a 500 m buffer zone appears to be sufficient to contain effects from releases in the wider countryside away from designated areas, whereas licenced releasing within designated areas and their buffers should reflect careful consideration of impact pathways for designated features. We found no evidence that released pheasants outside pens affected soil nutrient levels or coverage by AWI species at any distance.

The negative ecological effects on woodland vascular plant species that could be attributed to the actions and eutrophication of released pheasants appear to be limited both in their form and the distance from the release pen. This is in contrast to a range of negative ecological effects that are clearly detected occurring within the release pen itself (reviewed in Madden and Sage 2020). Outside the pen, the physical actions of the birds, scraping the ground or wood whilst foraging might reduce decayed wood availability or the germinating seedlings and saplings needed for the regeneration of trees, or decrease overall plant species richness. Such effects do not appear to extend beyond 500 m from the release sites. Outside the pen, pheasants do not appear to change soil nutrient levels or deplete AWI species coverage. However, these negative ecological effects may occur outside pens at areas where keepers deliberately concentrate birds, such as at feeders or in game covers along woodland edges or hedgerows, that we intentionally did not survey. Therefore, our results should be interpreted as depicting the consequences of unmanaged dispersal by released pheasants in English and Welsh ASNW.

## Author Contributions

**Joah R. Madden:** conceptualization (equal), data curation (equal), formal analysis (lead), writing – original draft (lead), writing – review and editing (equal). **Maureen I. A. Woodburn:** investigation (equal), methodology (equal). **Clive E. Bealey:** investigation (equal), methodology (equal). **Joseph L. Werling:** investigation (equal), methodology (equal). **Alex N. Banks:** conceptualization (equal), formal analysis (supporting), funding acquisition (lead), methodology (equal), project administration (equal), writing – review and editing (equal). **Dan Abrahams:** methodology (equal). **Rufus B. Sage:** conceptualization (equal), data curation (supporting), formal analysis (supporting), investigation (equal), project administration (equal), writing – review and editing (equal).

## Funding

This work was supported by the Department for Environment, Food and Rural Affairs, UK Government.

## Conflicts of Interest

The work was funded by DEFRA to contribute evidence to inform licencing of gamebird releases in England. M.I.A.W., J.L.W., and R.B.S. work for the Game and Wildlife Conservation Trust, a charity that uses science to promote game and wildlife management as an essential part of nature conservation.

## Supporting information


**Data S1:** ece373170‐sup‐0001‐DataS1.zip.

## Data Availability

The data supporting this MS and the R code used in the analyses are available on Dryad Dataset https://doi.org/10.5061/dryad.cjsxksnmn.

## References

[ece373170-bib-0001] Aebischer, N. J. 2019. “Fifty‐Year Trends in UK Hunting Bags of Birds and Mammals, and Calibrated Estimation of National Bag Size, Using GWCT'S National Gamebag Census.” European Journal of Wildlife Research 65, no. 4: 64.

[ece373170-bib-0002] Alsop, J. , and E. Goldberg . 2018. Synthesis of Evidence and Statement of Rationale: Cessation of Pheasant (Phasianus Colchicus) Feeding and Game Driving Activities Within Meadow Place Wood on the Derbyshire Dales NNR. Natural England Report NE2018‐DDNNR‐MPW‐PE003.

[ece373170-bib-0003] Brooks, M. E. , K. Kristensen , K. J. Van Benthem , et al. 2017. “glmmTMB Balances Speed and Flexibility Among Packages for Zero‐Inflated Generalized Linear Mixed Modeling.” R Journal 9: 378–400. 10.32614/RJ-2017-066.

[ece373170-bib-0004] Callegari, S. E. 2006. The Impact of Released Gamebirds on the Nature Conservation Value of Chalk Grassland in Central Southern Britain, PhD Thesis. University of Reading.

[ece373170-bib-0005] Capstick, L. A. , R. A. H. Draycott , C. M. Wheelwright , D. E. Ling , R. B. Sage , and A. N. Hoodless . 2019. “The Effect of Game Management on the Conservation Value of Woodland Rides.” Forest Ecology and Management 454: 117242.

[ece373170-bib-0006] Capstick, L. A. , R. B. Sage , and A. Hoodless . 2019. “Ground Flora Recovery in Disused Pheasant Pens Is Limited and Affected by Pheasant Release Density.” Biological Conservation 231: 181–188.

[ece373170-bib-0007] Dahl, E. , and E. Hadac . 1941. “Strandgesellschaften der Insel Ostøy im Oslofjord. Eine flanzensoziologische Studie.” Nytt Magasin Fur Naturvidenskapene B 82: 251–312.

[ece373170-bib-0008] Draycott, R. A. , A. N. Hoodless , and R. B. Sage . 2008. “Effects of Pheasant Management on Vegetation and Birds in Lowland Woodlands.” Journal of Applied Ecology 45, no. 1: 334–341.

[ece373170-bib-0009] Game Conservancy Trust . 1996. Gamebird Releasing. Game Conservancy Ltd.

[ece373170-bib-0010] Gilbert, J. 2007. National Inventory of Woodland and Trees (1995–1999): Analysis of Management and Biodiversity Data. Forest Research, Forestry Commission.

[ece373170-bib-0011] Grime, J. P. 2006. Plant Strategies, Vegetation Processes, and Ecosystem Properties. John Wiley & Sons.

[ece373170-bib-0012] Hall, A. , R. A. Sage , and J. R. Madden . 2021. “The Effects of Released Pheasants on Invertebrate Populations in and Around Woodland Release Sites.” Ecology and Evolution 11, no. 19: 13559–13569. 34646489 10.1002/ece3.8083 PMC8495776

[ece373170-bib-0013] Hartig, F. 2022. DHARMa: Residual Diagnostics for Hierarchical (Multi‐Level/Mixed) Regression Models. https://CRAN.R‐project.org/package=DHARMa.

[ece373170-bib-0014] Hermy, M. , O. Honnay , L. Firbank , C. Grashof‐Bokdam , and J. E. Lawesson . 1999. “An Ecological Comparison Between Ancient and Other Forest Plant Species of Europe, and the Implications for Forest Conservation.” Biological Conservation 91, no. 1: 9–22.

[ece373170-bib-0015] Heydon, M. J. , and J. C. Reynolds . 2000. “Demography of Rural Foxes (*Vulpes vulpes*) in Relation to Cull Intensity in Three Contrasting Regions of Britain.” Journal of Zoology 251, no. 2: 265–276.

[ece373170-bib-0016] Hill, D. A. 2005. Handbook of Biodiversity Methods: Survey, Evaluation and Monitoring. Cambridge University Press.

[ece373170-bib-0017] Hill, D. A. , and P. A. Robertson . 1988. The Pheasant: Ecology, Management, and Conservation. BSP Professional Books.

[ece373170-bib-0018] Hill, M. O. , J. O. Mountford , D. B. Roy , and R. G. H. Bunce . 1999. Ellenberg's Indicator Values for British Plants. ECOFACT Volume 2 Technical Annex. Institute of Terrestrial Ecology. http://nora.nerc.ac.uk/id/eprint/6411/.

[ece373170-bib-0019] Hipps, N. A. , M. J. Davies , P. Dodds , and G. P. Buckley . 2005. “The Effects of Phosphorus Nutrition and Soil pH on the Growth of Some Ancient Woodland Indicator Plants and Their Interaction With Competitor Species.” Plant and Soil 271: 131–141.

[ece373170-bib-0020] Humphrey, J. W. , A. L. Sippola , G. Lempérière , B. Dodelin , K. N. A. Alexander , and J. E. Butler . 2005. “Deadwood as an Indicator of Biodiversity in European Forests: From Theory to Operational Guidance.” Monitoring and Indicators of Forest Biodiversity in Europe–From Ideas to Operationality 51: 193–206.

[ece373170-bib-0021] Kirby, K. J. , S. M. Smart , H. I. J. Black , R. G. H. Bunce , P. M. Corney , and R. J. Smithers . 2005. Long‐Term Ecological Change in British Woodlands (1971–2001). English Nature Research Reports No. 653. English Nature.

[ece373170-bib-0022] Lenth, R. 2025. emmeans: Estimated Marginal Means, aka Least‐Squares Means. https://rvlenth.github.io/emmeans/.

[ece373170-bib-0023] Long, P. R. , L. Petrokofsky , W. J. Harvey , P. Orsi , M. W. Jordon , and G. Petrokofsky . 2025. “Structural Diversity and Biodiversity of Forest and Hedgerow in Areas Managed for Pheasant Shooting Across the UK.” Forests 16, no. 8: 1249.

[ece373170-bib-0024] Low, P. , N. Low , and N. Owens . 2003. Study of Vegetation in the Pheasant Pen, Old Sulehay Forest. Sulehay Nature Reserve.

[ece373170-bib-0025] Madden, J. R. 2021. “How Many Gamebirds Are Released in the UK Each Year?” European Journal of Wildlife Research 67, no. 4: 72.

[ece373170-bib-0026] Madden, J. R. 2023. A Review of the Ecological Effects of Gamebird Release and Management in Wales, 75. Natural Resources Wales.

[ece373170-bib-0027] Madden, J. R. , A. Hall , and M. A. Whiteside . 2018. “Why Do Many Pheasants Released in the UK Die, and How Can We Best Reduce Their Natural Mortality?” European Journal of Wildlife Research 64, no. 4: 40. 32214945 10.1007/s10344-018-1199-5 PMC7088407

[ece373170-bib-0028] Madden, J. R. , and R. B. Sage . 2020. Ecological Consequences of Gamebird Releasing and Management on Lowland Shoots in England: A Review by Rapid Evidence Assessment for Natural England and the British Association of Shooting and Conservation. Natural England Evidence Review NEER016.

[ece373170-bib-0029] Mason, L. R. , J. E. Bicknell , J. Smart , and W. J. Peach . 2020. The Impacts of Non‐Native Gamebird Release in the UK: An Updated Evidence Review. RSPB Research Report No. 66. RSPB Centre for Conservation Science.

[ece373170-bib-0030] Minter, M. , L. R. Mason , M. D. Burgess , W. J. Peach , and J. Hughes . 2024. “Understanding Stakeholder Perceptions on the Impacts of Gamebird Releasing on or Near UK Protected Sites.” Journal for Nature Conservation 78: 126581.

[ece373170-bib-0031] Neumann, J. L. , G. J. Holloway , R. B. Sage , and A. N. Hoodless . 2015. “Releasing of Pheasants for Shooting in the UK Alters Woodland Invertebrate Communities.” Biological Conservation 191: 50–59.

[ece373170-bib-0032] Oldfield, T. E. E. , R. I. Smith , A. R. Harrop , and N. Leader‐Williams . 2003. “Field Sports and Conservation in the United Kingdom.” Nature 423: 531–533. 12774120 10.1038/nature01678

[ece373170-bib-0033] Peterken, G. F. 2000. “Identifying Ancient Woodland Using Vascular Plant Indicators.” British Wildlife 11: 153–158.

[ece373170-bib-0034] Powolny, T. , and A. Czajkowski . 2022. Conservation and Management of Game Birds in Europe. Species of Annex II/A of the Birds Directive. OMPO.

[ece373170-bib-0035] Pressland, C. L. 2009. The Impact of Releasing Pheasants for Shooting on Invertebrates in British Woodlands. PhD Thesis. University of Bristol.

[ece373170-bib-0036] R Core Team . 2024. R: A Language and Environment for Statistical Computing. R Foundation for Statistical Computing. https://www.r‐project.org/.

[ece373170-bib-0037] Rackham, O. 2003. Ancient Woodland: Its History, Vegetation and Uses in England. Castlepoint Press.

[ece373170-bib-0038] Robertson, P. A. , M. I. A. Woodburn , and D. A. Hill . 1993. “Factors Affecting Winter Pheasant Density in British Woodlands.” Journal of Applied Ecology 30: 459–464.

[ece373170-bib-0039] Rothero, G. 2006. Baseline Surveys of Tortula Leucostoma and *Athalamia hyalina* on Craig Leek SSSI, 176. Scottish Natural Heritage.

[ece373170-bib-0040] Sage, R. B. 2018. Impacts of Pheasant Releasing for Shooting on Habitats and Wildlife on the South Exmoor Estates. GWCT report.

[ece373170-bib-0041] Sage, R. B. , J. Brewin , D. C. Stevens , and R. A. H. Draycott . 2021. Gamebird Releasing and Management in the UK. A Review of Ecological Considerations, Best Practice Management and Delivering Net Biodiversity Gain. Game & Wildlife Conservation Trust.

[ece373170-bib-0042] Sage, R. B. , A. N. Hoodless , M. I. A. Woodburn , R. A. H. Draycott , J. R. Madden , and N. W. Sotherton . 2020. “Summary Review and Synthesis—Effects on Habitats and Wildlife of the Release and Management of Pheasants and Red‐Legged Partridges on UK Lowland Shoots.” Wildlife Biology 2020: 1–12.

[ece373170-bib-0043] Sage, R. B. , C. Ludolf , and P. A. Robertson . 2005. “The Ground Flora of Ancient Semi‐Natural Woodlands in Pheasant Release Pens in England.” Biological Conservation 122, no. 2: 243–252.

[ece373170-bib-0044] Sage, R. B. , M. I. Woodburn , and J. R. Coomes . 2025. “Seasonal Densities of Released Common Pheasants *Phasianus colchicus* and Red‐Legged Partridges *Alectoris rufa* on Land Used for Shooting and on Nearby Non‐Release Land in Southern England.” Bird Study 72, no. 3: 1–15.

[ece373170-bib-0045] Sage, R. B. , M. I. A. Woodburn , R. A. H. Draycott , A. N. Hoodless , and S. Clarke . 2009. “The Flora and Structure of Farmland Hedges and Hedgebanks Near to Pheasant Release Pens Compared With Other Hedges.” Biological Conservation 142, no. 7: 1362–1369.

[ece373170-bib-0046] Smith, P. 2014. Effects of Fixed Nitrogen Eutrophication on Lichen Floras – Does Pheasant Rearing Represent a Threat to the Lichen Flora of Croxton Park? Natural England Technical report.

[ece373170-bib-0047] Turner, C. 2007. The Fate and Management of Pheasants ( *Phasianus colchicus* ) Released in the UK. University of London.

[ece373170-bib-0048] Woodburn, M. , and R. Sage . 2005. Effect of Pheasant Releasing on Edge Habitats, 36–37. Game and Wildlife Conservation Trust Review of 2004.

