# Supplementary material for: An Evaluation of the Distance at Which Direct Ecological Effects of Released Pheasants Extend Beyond Their Release Sites
Source: Ecol Evol. 2026 Mar 4;16(3):e73170. doi: 10.1002/ece3.73170 (PMC12959934; doi:10.1002/ece3.73170)
Supplement: Supplementary file 1 — Data S1: ece373170‐sup‐0001‐DataS1.zip. [file ECE3-16-e73170-s001.zip › ece373170-sup-0001-ESM1.docx]

**An evaluation of the distance at which direct ecological effects of released pheasants extend beyond their release sites**

Joah R. Madden^1^, Maureen I. A. Woodburn^2^, Clive E. Bealey^3^, Joseph L. Werling^2^, Alex N. Banks^4^, Dan Abrahams^4^ and Rufus B. Sage^2^

**Details of the soil chemical analysis procedure as provided by the University of Exeter**

**Potassium Analysis by ICP-OES.**

*Sample preparation - digestion*

Equipment

Milestone Ethos Easy advanced microwave digester with MAXI-44 rotor and TMF vessels, lids, and caps.

Balance - 4 decimal place

Brand set volume concentrated acid dispenser

Pipette variable volume

Volumetric flasks 50ml

Plastic funnels and filter papers

Plastic centrifuge tubes 50ml

Wash bottle Deionised water

Concentrated Nitric acid – Fisher AR 37%

Concentrated Hydrochloric acid – Fisher AR 70%

Certified Reference material (CRM2) - Sigma-Aldrich Metals in Soil SQ001-30G lot. LRAC6625.

Reference 2C – Soil sample 2C as supplied by the client.

Dried soil samples – analysed as sent by the client to the laboratory

Neutracon cleaning bath 10% for vessel decontamination and cleaning

*Sample preparation - methodology*

~ 0.5g of the samples as presented by the client to the laboratory, were directly weighed into microwave digest vessels. Masses (mg) were recorded to 4 decimal places.

Concentrated acid reverse Aqua regia of ratio 3:1, 9ml Nitric and 3ml Hydrochloric acid was added to the samples and then digested in a closed vessel Microwave (Milestone Ethos Easy advanced microwave digester with MAXI-44 rotor).

The acid digested samples were then gravity filtered into 50ml Standard Volumetric flasks and made up to volume using deionised water. The digested samples were then transferred into 50ml plastic centrifuge tubes for storage in the cold store (4^o^C).

A Certified Reference Material (CRM2) Sigma-Aldrich Metals in Soil, SQ001-30G lot no. LRAC6625 was digested with the first microwaved sample batch.

Sample (2C) (as supplied by the client) was repeat analysed as a reference and was redigested alongside each batch of microwave digested samples. n=20 samples, 1 x blank, 1 x CRM (or 1 x reference sample) were digested in each microwave-prepared batch of samples.

Microwave digest vessels were decontaminated between digest batches in Neutracon 10% bath and rinsed thoroughly in deionised water.

The digested samples were then analysed for Potassium using ICP-OES (Agilent ICP-OES 5110 with autosampler)

**Phosphorous Analysis**

Equipment

Agilent ICP-OES 5110 instrument and autosampler

Variable volume pipettes and tips

Brand set volume concentrated acid dispenser

Volumetric flasks 100ml

Concentrated Nitric acid

Wash bottle Deionised water

Phosphorus suppliers stock standard – Sigma-Aldrich 102262059 Phosphorus Standard for ICP 1000ppm, Lot. BCCD2573

Multi element suppliers stock standard – Sigma-Aldrich 1.11355.0100 ICP multi element standard solution IV 1000ppm. Lot no. HC15457555

Sample acid digests

Instrument calibration standards were prepared in 100ml volumetric flasks from supplier stock standards. Concentrated Nitric acid was added to a volume of deionised water in the flasks to produce a 2% acid. The Phosphorus and multi-element stock standards were added to the flasks and made up to the final volume with deionised water.

The ICP-OES was calibrated using the standards and the sample acid digests were analysed without initial dilution.

The data results from the ICP-OES analysis are presented in parts per million (ppm).
