# Supplementary material for: An Evaluation of the Distance at Which Direct Ecological Effects of Released Pheasants Extend Beyond Their Release Sites
Source: Ecol Evol. 2026 Mar 4;16(3):e73170. doi: 10.1002/ece3.73170 (PMC12959934; doi:10.1002/ece3.73170)
Supplement: Supplementary file 1 — Data S1: ece373170‐sup‐0001‐DataS1.zip. [file ECE3-16-e73170-s001.zip › ece373170-sup-0002-ESM2.docx]

**ESM2:** **An evaluation of the distance at which direct ecological effects of released pheasants extend beyond their release sites**

Joah R. Madden^1^, Maureen I. A. Woodburn^2^, Clive E. Bealey^3^, Joseph L. Werling^2^, Alex N. Banks^4^, Dan Abrahams^4^ and Rufus B. Sage^2^

**Species included as Ancient Woodland Indicators**

| Apetalous sandwort | *Moehringia trinervia* |
| --- | --- |
| Barren strawberry | *Potentilla sterilis* |
| Bilberry | *Vaccinium myrtillus* |
| Bittercress | *Cardamine hirsute [??]* |
| Black bryony | *Dioscorea communis* |
| Buckler fern | *Dryopteris dilatata* |
| Bush vetch | *Vicia sepium* |
| Butcher's-broom | *Ruscus aculeatus* |
| Cow wheat | *Melampyrum [spp??]* |
| Early dog violet | *Viola reichenbachiana* |
| Golden rod | *Solidago virgaurea [??]* |
| Great woodrush | *Luzula sylvatica* |
| Hairy brome | *Bromus ramosus* |
| Hairy woodrush | *Luzula acuminata* |
| Hard fern | *Blechnum spicant* |
| Herb-Paris | *Paris quadrifolia* |
| Moschatel | *Adoxa moschatellina* |
| Pendulous sedge | *Carex pendula* |
| Pignut | *Conopodium majus* |
| Primrose | *Primula vulgaris* |
| Spurge-laurel | *Daphne laureola* |
| Wild currant | *Ribes spicatum* |
| Wild garlic | *Allium ursinum* |
| Wood anemone | *Anemonoides nemorosa* |
| Wood meadow grass | *Poa nemoralis* |
| Wood melick | *Melica uniflora* |
| Wood sedge | *Carex sylvatica* |
| Wood sorrel | *Oxalis acetosella [??]* |
| Wood speedwell | *Veronica montana* |
| Yellow archangel | *Lamium galeobdolon* |
| Yellow pimpernel | *Lysimachia nemorum* |

Deliberately excluded:

Bluebell *Hyacinthoides non-scripta* (confusion with invasive Spanish bluebells *Hyacinthoides hispanica*)

Dog Mercury *Mercurialis perennis* (prolific in S England and not AWI there)

**Species included as Weeds**

| Cleavers | *Galium aparine* |
| --- | --- |
| Burdock | *Arctium lappa [??]* |
| Chickweed | *Stellaria media* |
| Clover | *Trifolium [spp??]* |
| Dandelion | *Taraxacum officinale* |
| Dock | *Rumex [spp??]* |
| Ground Ivy | *Glechoma hederacea* |
| Hedge Woundwort | *Stachys sylvatica* |
| Hemp nettle | *Galeopsis tetrahit* |
| Hogweed | *Heracleum sphondylium* |
| Ragwort | *Jacobaea vulgaris* |
| Rough Meadow Grass | *Poa trivialis* |
| Selfheal | *Prunella vulgaris* |
| Soft Rush | *Juncus effusus* |
| Stinging nettle | *Urtica dioica* |
