# Supplementary material for: An Evaluation of the Distance at Which Direct Ecological Effects of Released Pheasants Extend Beyond Their Release Sites
Source: Ecol Evol. 2026 Mar 4;16(3):e73170. doi: 10.1002/ece3.73170 (PMC12959934; doi:10.1002/ece3.73170)
Supplement: Supplementary file 1 — Data S1: ece373170‐sup‐0001‐DataS1.zip. [file ECE3-16-e73170-s001.zip › ece373170-sup-0003-ESM3.docx]

**ESM3:** **An evaluation of the distance at which direct ecological effects of released pheasants extend beyond their release sites**

Joah R. Madden^1^, Maureen I. A. Woodburn^2^, Clive E. Bealey^3^, Joseph L. Werling^2^, Alex N. Banks^4^, Dan Abrahams^4^ and Rufus B. Sage^2^

**Full model outputs**

1. **Do the numbers of gamebirds detected differ depending on distance from the pen?**

|  | Estimate | Std. Error | z value |
| --- | --- | --- | --- |
| (Intercept – 10m survey point/Winter) | 1.82 | 0.23 | 7.92 |
| 100m survey point | -0.29 | 0.27 | -1.04 |
| 250m survey point | -0.65 | 0.28 | -2.29 |
| 500m survey point | -1.36 | 0.31 | -4.39 |
| 1000m survey point/Control | -2.11 | 0.36 | -5.91 |
| Summer | -2.30 | 0.37 | -6.21 |
| 100m survey point x Summer | -0.10 | 0.55 | -0.18 |
| 250m survey point x Summer | 1.04 | 0.51 | 2.05 |
| 500m survey point x Summer | 1.30 | 0.55 | 2.39 |
| 1000m survey point/Control x Summer | 1.99 | 0.58 | 3.44 |

1. **Do soil nutrient levels differ depending on distance from the pen?**

| 1. Nitrates | Estimate | Std. Error | z value |
| --- | --- | --- | --- |
| (Intercept – 10m survey point) | 2.96 | 0.45 | 6.55 |
| 100m survey point | -0.63 | 0.51 | -1.25 |
| 250m survey point | 0.21 | 0.51 | 0.41 |
| 500m survey point | -0.13 | 0.51 | -0.25 |
| 1000m survey point/Control | 0.29 | 0.51 | 0.57 |
| 1. Phosphates | Estimate | Std. Error | z value |
| (Intercept – 10m survey point) | 0.09 | 0.02 | 3.66 |
| 100m survey point | -0.01 | 0.02 | -0.43 |
| 250m survey point | -0.01 | 0.02 | -0.60 |
| 500m survey point | -0.02 | 0.02 | -0.99 |
| 1000m survey point/Control | 0.01 | 0.02 | 0.42 |
| 1. Potassium | Estimate | Std. Error | z value |
| (Intercept – 10m survey point) | 2.47 | 0.28 | 8.68 |
| 100m survey point | -0.06 | 0.18 | -0.35 |
| 250m survey point | 0.01 | 0.18 | 0.04 |
| 500m survey point | 0.17 | 0.18 | 0.94 |
| 1000m survey point/Control | 0.37 | 0.18 | 2.03 |

1. **Does the coverage of bare ground differ depending on distance from the pen?**

|  | Estimate | Std. Error | z value |
| --- | --- | --- | --- |
| (Intercept – 10m survey point) | 3.42 | 0.14 | 24.54 |
| 100m survey point | -0.14 | 0.15 | -0.96 |
| 250m survey point | 0.05 | 0.15 | 0.31 |
| 500m survey point | -0.02 | 0.15 | -0.14 |
| 1000m survey point/Control | 0.00 | 0.14 | -0.03 |

1. **Does the presence of germinating seedlings and saplings differ depending on distance from the pen?**

|  | Estimate | Std. Error | z value |
| --- | --- | --- | --- |
| (Intercept – 10m survey point) | -1.15 | 0.31 | -3.69 |
| 100m survey point | -0.06 | 0.34 | -0.17 |
| 250m survey point | 0.38 | 0.33 | 1.15 |
| 500m survey point | 0.63 | 0.33 | 1.93 |
| 1000m survey point/Control | 1.11 | 0.33 | 3.40 |

1. **Does the availability of decayed wood differ depending on distance from the pen?**

|  | Estimate | Std. Error | z value |
| --- | --- | --- | --- |
| (Intercept – 10m survey point) | 3.10 | 0.19 | 16.73 |
| 100m survey point | 0.05 | 0.15 | 0.31 |
| 250m survey point | 0.02 | 0.16 | 0.12 |
| 500m survey point | 0.13 | 0.16 | 0.83 |
| 1000m survey point/Control | 0.62 | 0.16 | 3.95 |

1. **Does the average plant community N sensitivity in a quadrat differ depending on distance from the pen?**

|  | Estimate | Std. Error | z value |
| --- | --- | --- | --- |
| (Intercept – 10m survey point) | 5.63 | 0.14 | 39.76 |
| 100m survey point | -0.07 | 0.09 | -0.77 |
| 250m survey point | -0.05 | 0.08 | -0.61 |
| 500m survey point | 0.28 | 0.09 | 3.33 |
| 1000m survey point/Control | 0.06 | 0.08 | 0.74 |

1. **Does the abundance of Ancient Woodland Indicator species differ with distance from the pen?**

|  | Estimate | Std. Error | z value |
| --- | --- | --- | --- |
| (Intercept – 10m survey point) | 3.05 | 0.20 | 15.12 |
| 100m survey point | -0.09 | 0.22 | -0.40 |
| 250m survey point | 0.02 | 0.22 | 0.10 |
| 500m survey point | -0.06 | 0.22 | -0.26 |
| 1000m survey point/Control | -0.33 | 0.22 | -1.47 |

1. **Does the abundance of weed species differ with distance from the pen?**

|  | Estimate | Std. Error | z value |
| --- | --- | --- | --- |
| (Intercept – 10m survey point) | 1.10 | 0.50 | 2.18 |
| 100m survey point | -0.65 | 0.47 | -1.38 |
| 250m survey point | -0.48 | 0.48 | -1.01 |
| 500m survey point | 0.82 | 0.46 | 1.77 |
| 1000m survey point/Control | 0.77 | 0.52 | 1.47 |

1. **Does the number of vascular plant species differ with distance from the pen?**

|  | Estimate | Std. Error | z value |
| --- | --- | --- | --- |
| (Intercept – 10m survey point) | 1.25 | 0.08 | 15.79 |
| 100m survey point | 0.05 | 0.07 | 0.73 |
| 250m survey point | 0.04 | 0.07 | 0.48 |
| 500m survey point | 0.25 | 0.07 | 3.51 |
| 1000m survey point/Control | 0.25 | 0.07 | 3.64 |
